# Unraveling the pathogenic potential of the *Pentatrichomonas hominis* PHGD strain: impact on IPEC-J2 cell growth, adhesion, and gene expression[Fn FN1]

**DOI:** 10.1051/parasite/2024014

**Published:** 2024-03-26

**Authors:** Yibin Zhu, Haiming Cai, Siyun Fang, Hanqin Shen, Zhuanqiang Yan, Dingai Wang, Nanshan Qi, Juan Li, Minna Lv, Xuhui Lin, Junjing Hu, Yongle Song, Xiangjie Chen, Lijun Yin, Jianfei Zhang, Shenquan Liao, Mingfei Sun

**Affiliations:** 1 Key Laboratory of Livestock Disease Prevention of Guangdong Province, Key Laboratory of Avian Influenza and Other Major Poultry Diseases Prevention and Control, Ministry of Agriculture and Rural Affairs, Institute of Animal Health, Guangdong Academy of Agricultural Sciences Guangzhou 510640 China; 2 Wen’s Group Academy, Wen’s Foodstuffs Group Co., Ltd. Xinxing Guangdong 527400 China; 3 Guangdong Jingjie Inspection and Testing Co., Ltd. Xinxing Guangdong 527400 China

**Keywords:** *Pentatrichomonas hominis*, IPEC-J2, cell viability, inflammatory response

## Abstract

*Pentatrichomonas hominis*, a flagellated parasitic protozoan, predominantly infects the mammalian digestive tract, often causing symptoms such as abdominal pain and diarrhea. However, studies investigating its pathogenicity are limited, and the mechanisms underlying *P. hominis*-induced diarrhea remain unclear. Establishing an *in vitro* cell model for *P. hominis* infection is imperative. This study investigated the interaction between *P. hominis* and IPEC-J2 cells and its impact on parasite growth, adhesion, morphology, and cell viability. Co-cultivation of *P. hominis* with IPEC-J2 cells resulted in exponential growth of the parasite, with peak densities reaching approximately 4.8 × 10^5^ cells/mL and 1.2 × 10^6^ cells/mL at 48 h for initial inoculation concentrations of 10^4^ cells/mL and 10^5^ cells/mL, respectively. The adhesion rate of *P. hominis* to IPEC-J2 cells reached a maximum of 93.82% and 86.57% at 24 h for initial inoculation concentrations of 10^4^ cells/mL and 10^5^ cells/mL, respectively. Morphological changes in IPEC-J2 cells co-cultivated with *P. hominis* were observed, manifesting as elongated and irregular shapes. The viability of IPEC-J2 cells exhibited a decreasing trend with increasing *P. hominis* concentration and co-cultivation time. Additionally, the mRNA expression levels of IL-6, IL-8, and TNF-α were upregulated, whereas those of CAT and CuZn-SOD were downregulated. These findings provide quantitative evidence that *P. hominis* can promote its growth by adhering to IPEC-J2 cells, inducing morphological changes, reducing cell viability, and triggering inflammatory responses. Further *in vivo* studies are warranted to confirm these results and enhance our understanding of *P. hominis* infection.

## Abstract (fr)

*Pentatrichomonas hominis*, un protozoaire parasite flagellé, infecte principalement le tube digestif des mammifères, provoquant souvent des symptômes tels que des douleurs abdominales et de la diarrhée. Cependant, les études portant sur sa pathogénicité sont limitées et les mécanismes sous-jacents à la diarrhée induite par *P. hominis* restent flous. L’établissement d’un modèle cellulaire *in vitro* de l’infection à *P. hominis* est impératif. Cette étude a examiné l’interaction entre *P. hominis* et les cellules IPEC-J2 et son impact sur la croissance du parasite, l’adhésion, la morphologie et la viabilité cellulaire. La co-culture de *P. hominis* avec des cellules IPEC-J2 a entraîné une croissance exponentielle du parasite, avec des densités maximales atteignant environ 4,8 × 10^5^ cellules/mL et 1,2 × 10^6^ cellules/mL à 48 h pour des concentrations d’inoculation initiales de 10^4^ cellules/mL et 10^5^ cellules/mL, respectivement. Le taux d’adhésion de *P. hominis* aux cellules IPEC-J2 a atteint un maximum de 93,82 % et 86,57 % après 24 h pour des concentrations d’inoculation initiales de 10^4^ cellules/mL et 10^5^ cellules/mL, respectivement. Des changements morphologiques dans les cellules IPEC-J2 co-cultivées avec *P. hominis* ont été observés, se manifestant par des formes allongées et irrégulières. La viabilité des cellules IPEC-J2 a montré une tendance à la baisse avec l’augmentation de la concentration de *P. hominis* et de la durée de co-culture. De plus, les niveaux d’expression d’ARNm d’IL-6, d’IL-8 et de TNF-α étaient régulés positivement, tandis que ceux de CAT et de CuZn-SOD étaient régulés négativement. Ces résultats fournissent des preuves quantitatives que *P. hominis* peut favoriser sa croissance en adhérant aux cellules IPEC-J2, en induisant des changements morphologiques, en réduisant la viabilité cellulaire et en déclenchant des réponses inflammatoires. D’autres études *in vivo* sont nécessaires pour confirmer ces résultats et améliorer notre compréhension de l’infection à *P. hominis*.

## Introduction

Trichomonads are ancient eukaryotic protists that inhabit warm, moist, and anaerobic mucosal environments within vertebrate and invertebrate hosts [7]. While numerous studies have extensively investigated trichomonads, particularly focusing on *Trichomonas vaginalis* and its implications in the reproductive tract [27] (the causative agent of the most common nonviral sexually transmitted disease, trichomoniasis), mechanistic studies on trichomonads that infect the gastrointestinal tract are scarce. One notable pathogenic species in this context is *Pentatrichomonas hominis*, which causes gastrointestinal symptoms in both humans and animals. It has been isolated from the reproductive and intestinal tracts of bovines [25]. To understand the significance of these infections and develop effective treatment strategies, it is imperative to investigate the virulence factors and pathogenesis of diseases caused by trichomonads that infect the gastrointestinal tract.

*Pentatrichomonas hominis* is an anaerobic flagellated protozoan that primarily colonizes the large intestines of mammals and is primarily transmitted through the fecal-oral route [20]. Initially considered a commensal protozoan, *P. hominis* has been found to induce gastrointestinal symptoms, including diarrhea, in humans [1], dogs [24], and cats [3]. It has also been linked to systemic lupus erythematosus [23], irritable bowel syndrome [30], and rheumatoid arthritis [6] in humans. Notably, a significant number of *P. hominis* infections have been reported in Chinese patients with gastrointestinal cancer [36]. Given its zoonotic potential and pathogenic nature, numerous studies have been conducted to investigate its prevalence and pathogenicity in various vertebrates, including dogs, cattle, pigs, monkeys, and other animals. However, our understanding of *P. hominis* pathogenicity in animals, particularly in pigs, remains limited. Considering the economic importance of pigs in the food production industry and the potential risk of zoonotic transmission, it is essential to investigate the pathogenic potential of *P. hominis* in pigs for disease prevention, control strategies, and assessing the risk of transmission to humans.

In recent years, several studies have focused on developing *in vitro* infection models for trichomonads [18, 19, 31], to investigate their colonization and pathogenic mechanisms. These models serve as valuable tools for examining the establishment of trichomonads and the harm caused by them. The co-cultivation of trichomonads with intestinal porcine epithelial cells (IPEC-J2 cells), originally developed by the Gookin group using *T. foetus* (which also infects pigs) [33], served as an important foundation for our study. *Pentatrichomonas hominis* has been detected in the feces of piglets with diarrhea, posing a potential risk to both animal and public health. However, research on the dynamics of infection and pathogenicity of *P. hominis*, particularly within the epithelial cell system of the pig digestive tract, is limited. Therefore, this study aimed to establish an infection model for the PHGD strain of *P. hominis* in IPEC-J2 cells. The findings of this study will provide valuable insights into the epidemiology and control of *P. hominis* in pig populations.

## Materials and methods

### Parasite isolation and culture

The *P. hominis* PHGD strain used in this study was isolated from the feces of diarrheal piglets and preserved in the laboratory. The steps involved in this process are as follows: fresh fecal samples were collected and processed within 2 h of collection. The samples were diluted and washed in phosphate-buffered saline (PBS) to remove large particles. The filtrate was centrifuged at 500 × *g* for 10 min to concentrate the parasites and obtain pellets. Subsequently, the pellet was resuspended in a complete culture medium composed of Modified Diamond’s medium [12], supplemented with 10% fetal bovine serum (FBS), penicillin (100 IU/mL), and streptomycin (100 μg/mL). The parasites were subsequently cultured in 25 cm^2^ cell culture flasks at 37 °C under anoxic conditions.

### IPEC-J2 cell culture

Porcine IPEC-J2 intestinal cells were obtained from a commercial source and cultured following standard protocols [4]. The cells were cultured in a complete culture medium, consisting of Dulbecco’s Modified Eagle Medium/Nutrient Mixture F-12 (DMEM/F-12) (Gibco, USA) supplemented with 10% FBS (Gibco, USA) and 1% penicillin-streptomycin (100 U/mL). The cells were maintained at 37 °C with 5% CO_2_, and the culture medium was refreshed every 2–3 day. Subculturing was performed when the cells reached approximately 80% confluence.

### Co-cultivation of *P. hominis* with IPEC-J2 Cells

IPEC-J2 cells were seeded in 24-well cell culture plates at a density of 2.5 × 10^5^ cells/well and allowed to grow for 24 h to reach approximately 80% confluence. The *P. hominis* PHGD strain was harvested from the culture flask and resuspended in a complete culture medium. Different concentrations of parasites were introduced into the wells containing IPEC-J2 cells, including (1) a control group without parasites and (2) varying concentrations of parasites (e.g., 10^4^ cells/mL, 10^5^ cells/mL, and 10^6^ cells/mL). The plates were then incubated at 37 °C with 5% CO_2_ for the desired co-cultivation duration.

### Evaluation of *P. hominis* growth

To assess the growth of *P. hominis*, the parasite count was monitored at various time points during co-cultivation. Different concentrations of *P. hominis* PHGD were introduced to each group, including 10^4^ cells/mL, 10^5^ cells/mL, 10^6^ cells/mL, and a control group containing 10% FBS-supplemented DMEM/F-12 medium without IPEC-J2 cells. Following co-cultivation for specific durations of 12, 24, 36, 48, 60, and 72 h, the cells were treated with a 0.25% trypsin solution to release the parasites, and all parasites within each well were collected for enumeration. The collected samples were then centrifuged to concentrate the parasites, which were then resuspended in PBS. Enumeration of the parasites was performed using either a hemocytometer or an automated cell counter. To ensure the reliability and reproducibility of the results, each experimental condition was replicated three times.

### Morphological observations

The adhesion of *P. hominis* to IPEC-J2 cells was assessed through an adhesion assay. The cells were exposed to varying concentrations of *P. hominis* PHGD, including a control group treated with 1 mL of 10% FBS-supplemented DMEM/F-12 medium, alongside concentrations of 10^4^ cells/mL, 10^5^ cells/mL, 10^6^ cells/mL, 5 × 10^6^ cells/mL, and 10^7^ cells/mL. The co-cultivation period lasted for 6, 12, 18, and 24 h, during which the morphological characteristics of the cells were observed by using Leica DMi8 inverted fluorescence microscope (Leica Microsystems, Wetzlar, Germany) and documented through photography.

### Adhesion assay

The adhesion rate of the *P. hominis* PHGD strain to IPEC-J2 cells was comprehensively determined in this study. The cells were exposed to varying concentrations of *P. hominis* PHGD, including a control group treated with 1 mL of 10% FBS-supplemented DMEM/F-12 medium, as well as concentrations of 10^4^ cells/mL, 10^5^ cells/mL, and 10^6^ cells/mL. Following co-cultivation for specific time intervals of 12, 24, 36, 48, 60, and 72 h, the complete culture medium containing non-adherent *P. hominis* was collected from each well (referred to as P1). Subsequently, the cells were digested using a 0.25% trypsin solution (Gibco, Waltham, MA, USA), and the entire digestion solution containing adherent *P. hominis* was collected from each well (referred to as P2). The number of parasites in each sample was quantified. The adhesion rate was then calculated using Equation (1), as follows:

(1) $$\mathrm{Adhesion}\;\mathrm{rate}\;=\;\frac{P1}{P1+P2}\times 100\%.$$

### Cell viability assessment

After co-cultivation with *P. hominis*, the viability of IPEC-J2 cells was evaluated using a cell viability assay. The cells were exposed to varying concentrations of *P. hominis* PHGD, encompassing a control group treated with 100 μL 10% FBS-supplemented DMEM/F-12 medium, as well as concentrations of 10^4^ cells/mL, 10^5^ cells/mL, 10^6^ cells/mL, 5 × 10^6^ cells/mL, and 10^7^ cells/mL. Following co-cultivation for 12, 18, and 24 h, the cells were thoroughly washed with sterile PBS to eliminate any adherent *P. hominis*. Subsequently, a Cell Counting Kit-8 (CCK-8) assay (Beyotime, Haimen, China) was performed in accordance with the manufacturer’s instructions, utilizing Varioskan LUX multimode microplate reader, with absorbance measured at 450 nm to assess cell viability. Each experiment was conducted in triplicate to ensure the reproducibility and reliability of the results.

### Gene expression analysis

**Figure S1:**
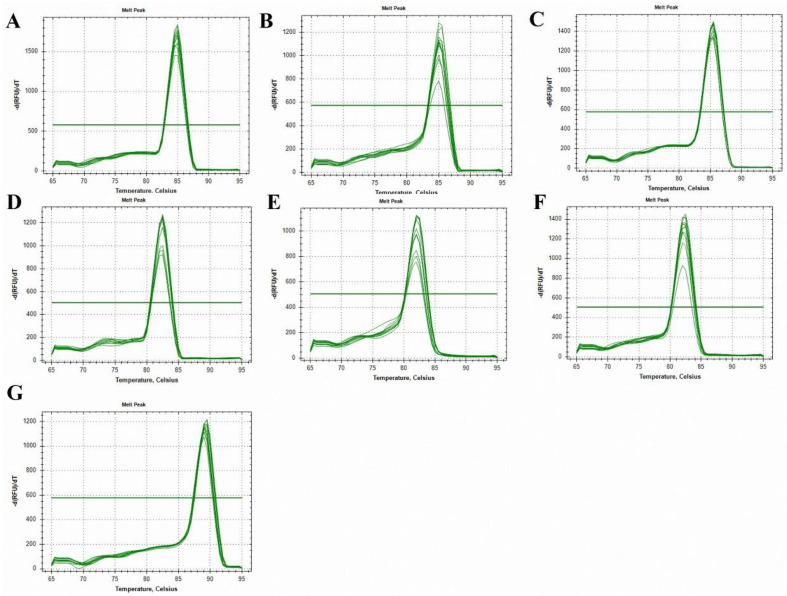
Melt curves of the target genes: CAT (A), CuZn-SOD (B), Mn-SOD (C), IL-6 (D), IL-8 (E), TNF-α (F), and β-actin (G).

The study aimed to investigate the regulatory effects of *P. hominis* PHGD on gene expression in IPEC-J2 cells by comparing two conditions: a control group treated with 1 mL 10% FBS-supplemented DMEM/F-12 medium and a treatment group exposed to a concentration of 10^6^ cells/mL. After an 18 h co-cultivation period, the cells underwent three washes with sterile PBS to eliminate any adherent *P. hominis*. Subsequently, RNA was extracted from the co-cultured cells using an EZNA Total RNA Kit I (Omega, Norcross, GA, USA), according to the manufacturer’s instructions. The extracted RNA was reverse transcribed into cDNA using an ABScript III RT Master Mix kit (ABclonal, Woburn, MA, USA). Quantitative PCR (qPCR) was performed to analyze the expression levels of target genes listed in Table 1. Figure S1 presents single melting curves, affirming the specificity of PCR products for all analyzed genes. The relative gene expression levels were calculated using the 2^−ΔΔCT^ method, employing β-actin as the reference genes. For qPCR analysis, the TB Green Premix Ex Taq II qPCR kit (Takara Bio Inc., Kusatsu, Japan) was employed, and fluorescence quantification was conducted using a Bio-Rad CFX Connect thermocycler (Bio-Rad, Irvine, CA, USA).

**Table 1:** Primers used for quantitative PCR in this study.

Gene	Prime ID	Primer sequences (5′ 3′)	Accession number	Source
CAT	CAT-F	CTTGGAACATTGTACCCGCT	NM_214301.2	[22]
	CAT-R	GTCCAGAAGAGCCTGAATGC		
CuZn-SOD	CuZn-SOD-F	CAGGTCCTCACTTCAATCC	NM_214301.2	[22]
	CuZn-SOD-R	CCAAACGACTTCCASCAT		
Mn-SOD	Mn-SOD-F	GGACAAATCTGAGCCCTAACG	NM_214127.2	[22]
	Mn-SOD-R	CCTTGTTGAAACCGAGCC		
IL-6	IL-6-F	ATCAGGAGACCTGCTTGATG	NM_214399.1	[37]
	IL-6-R	TGGTGGCTTTGTCTGGATTC		
IL-8	IL-8-F	TCCTGCTTTCTGCAGCTCTC	NM_213867.1	[37]
	IL-8-R	GGGTGGAAAGGTGTGGAATG		
TNF-α	TNF-α-F	CTGTAGGTTGCTCCCACCTG	NM_214022.1	[37]
	TNF-α-R	CCAGTAGGGCGGTTACAGAC		
β-actin	β-actin-F	GGACTTCGAGCAGGAGATGG	XM_003357928.4	[22]
	β-actin-R	GCACCGTGTTGGCGTAGAGG		

### Statistical analysis

All data obtained in this study were subjected to statistical analysis using IBM SPSS Statistics 19 software (IBM Corp., Armonk, NY, USA). Parametric data were analyzed using Student’s *t*-test or one-way analysis of variance (ANOVA), followed by a *post hoc* Holm-Sidak test for multiple comparisons. Nonparametric data were analyzed using the Mann–Whitney rank-sum test or Kruskal–Wallis ANOVA on ranks. The results are presented as means ± standard deviations (SD). A significance level of *p* < 0.05 was deemed statistically significant for all analyses. These statistical procedures were employed to ensure robust and reliable interpretation of the data, adhering to established scientific standards for significance testing.

## Results

### Growth curves of *P. hominis* PHGD strain in co-cultivation with IPEC-J2 cells

**Figure 1:**
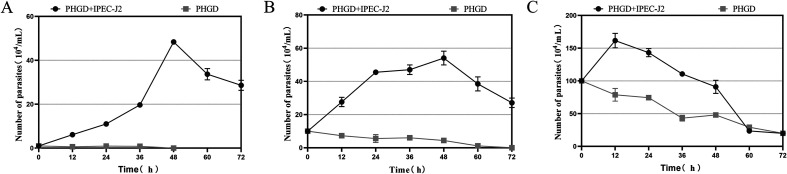
Growth curve of *P. hominis* co-incubated with IPEC-J2 cells. (A) depicts the growth curve with an initial inoculation concentration of 10^4^ cells/mL of *P. hominis*, while (B) illustrates the response to an initial inoculation of 10^5^ cells/mL of *P. hominis*. (C) shows the growth curve following an initial inoculation concentration of 10^6^ cells/mL of *P. hominis*. The *y*-axis represents the parasite count, and the *x*-axis denotes the incubation time in hours. The gray squares represent the growth curve results for individual *P. hominis*, while the black spheres depict the growth curve results when co-incubated with IPEC-J2 cells.

The co-cultivation of *P. hominis* PHGD strain with IPEC-J2 cells resulted in a notable enhancement in parasite growth. Figure 1 illustrates the growth curves of *P. hominis* at varying concentrations. In the co-cultivation group, the parasite exhibited exponential growth, reaching its peak at 48 h. In contrast, when cultured in isolation in 10% FBS-supplemented DMEM/F-12 medium, the parasite failed to exhibit any growth, and all parasites succumbed by the 48-h mark. The growth curves displayed variance depending on the initial inoculation concentration of *P. hominis* PHGD. In the co-cultivation group with an initial inoculation of 10^4^ cells/mL, the parasite demonstrated exponential growth in the first 48 h, followed by a decline. The peak parasite density reached approximately 4.8 × 10^5^ cells/mL at 48 h. For the co-cultivation group with an initial inoculation of 10^5^ cells/mL, the parasite density increased, reaching a peak of approximately 1.2 × 10^6^ cells/mL at 48 h.

### Adhesion rate of the *P. hominis* PHGD strain to IPEC-J2 cells

**Figure 2:**
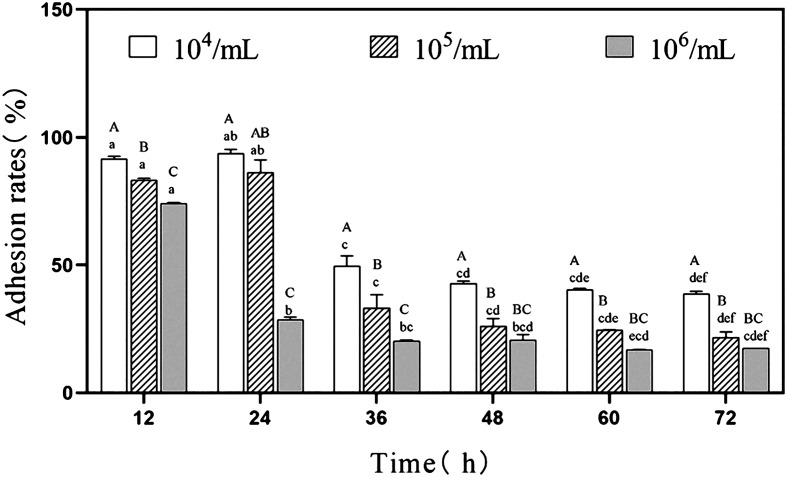
Adhesion rate of IPEC-J2 cells co-incubated with varying *P. hominis* concentrations. The adhesion rate is depicted as a bar graph, with the *y*-axis indicating the percentage of adhered cells, and the *x*-axis representing the incubation time in hours. Different colors and patterns represent the three initial inoculation amounts. To assess statistical significance, a one-way analysis of variance (ANOVA) was conducted. Letters A, B, and C indicate differences in initial inoculation amounts at the same time point, while lowercase letters a, b, c, d, e, and f denote differences in time points for the same initial inoculation amount. Similar letters indicate no significant difference.

Following the co-incubation of PHGD with IPEC-J2 cells, the adhesion of PHGD to IPEC-J2 cells was assessed, and the results are presented in Figure 2. The adhesion rate of PHGD to IPEC-J2 cells gradually decreased with the extension of co-incubation time and the increase of the initial inoculation amount. At 12 and 24 h, with an initial inoculation amount of 10^4^ cells/mL and 10^5^ cells/mL, the adhesion rate of PHGD to IPEC-J2 cells was significantly higher compared to other time points. The adhesion rate reached a maximum of 93.82% and 86.57% at 24 h following co-incubation for these two inoculation amounts, respectively. When the initial inoculation amount was 10^6^ cells/mL and incubated for 12 h, the adhesion rate of PHGD to IPEC-J2 cells was approximately 74.52%, which was significantly higher than that at other time points with the same inoculation concentration. At 12, 36, 48, 60, and 72 h, the adhesion rate of PHGD with an initial inoculation amount of 10^4^ cells/mL to IPEC-J2 cells was significantly higher than that at 10^5^ cells/mL and 10^6^ cells/mL.

### Morphological changes in IPEC-J2 cells induced by the *P. hominis* PHGD strain

**Figure 3:**
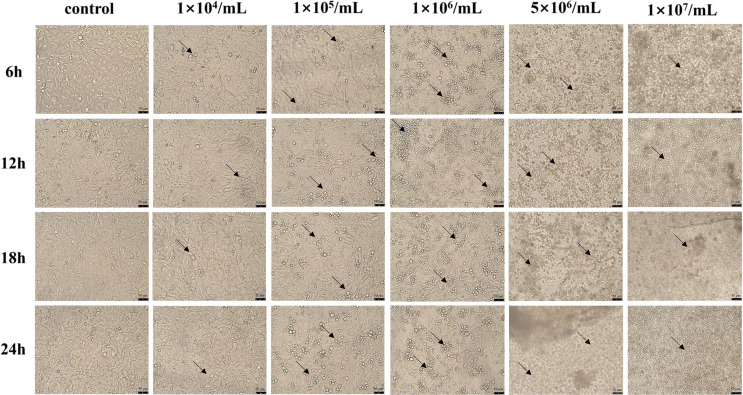
Morphological changes of IPEC-J2 cells after incubation with different concentrations of *P. hominis.* Microscopic images (200×) illustrating morphological alterations in IPEC-J2 cells co-incubated with varying concentrations (1 × 10^4^ cells/mL, 1 × 10^5^ cells/mL, 1 × 10^6^ cells/mL, 5 × 10^6^ cells/mL, 1 × 10^7^ cells/mL) of *P. hominis* (2.5 × 10^5^ IPEC-J2 cells) at different time points (6 h, 12 h, 18 h and 24 h). The black arrow marks the partially visible *P. hominis* parasites.

Co-cultivation with the *P. hominis* PHGD strain resulted in morphological changes in IPEC-J2 cells, as shown in Figure 3. Compared to the control group, the cells co-cultivated with *P. hominis* exhibited elongated and irregular shapes. The extent of these morphological changes increased with higher concentrations of *P. hominis*. At 24 h of co-cultivation, the cells in the control group maintained a typical cobblestone-like morphology, while the cells co-cultivated with *P. hominis* displayed elongated and stretched shapes. When IPEC-J2 cells were initially inoculated with 1 × 10^6^ cells/mL of *P. hominis*, significant proliferation of *P. hominis* was observed after 6 h of co-incubation, and the fusion of IPEC-J2 cells decreased after 18 h. In the case of initial inoculations of 5 × 10^6^ cells/mL and 1 × 10^7^ cells/mL of *P. hominis*, the high density of parasites after 6 h of co-incubation resulted in a significant number of cell deaths. These morphological changes indicate that the *P. hominis* PHGD strain can indeed impact the morphology of IPEC-J2 cells.

### Viability of IPEC-J2 cells following co-cultivation with the *P. hominis* PHGD strain

**Figure 4:**
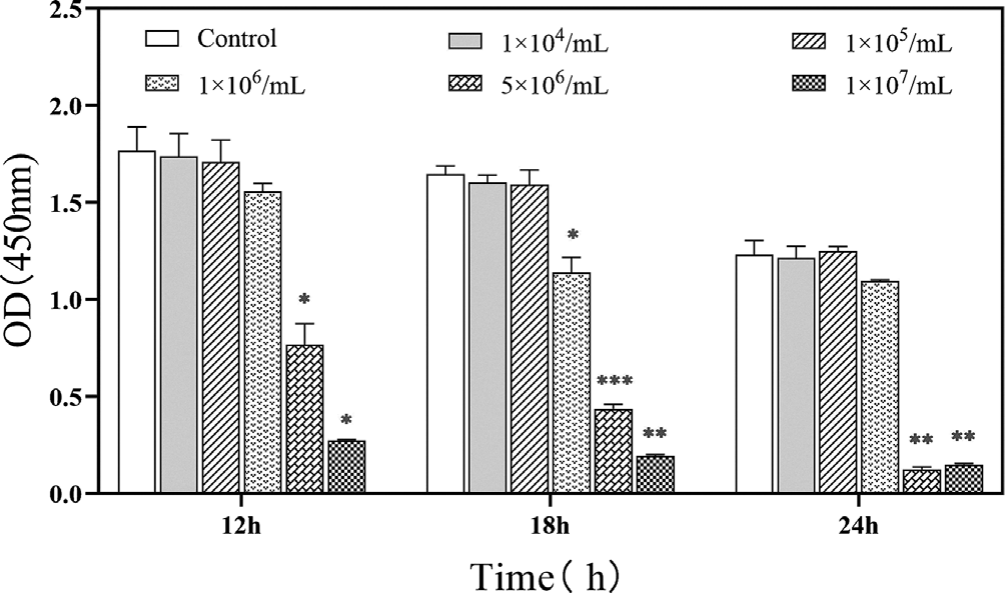
Determination of cell viability of IPEC-J2 cells induced by varying concentrations of *P. hominis.* Cell viability was monitored by using CCK8 assay kits, in which optical density (OD) 450 nm values represent cell viability. The *y*-axis denotes the OD450 nm, and the *x*-axis indicates the various *P. hominis* co-culture time points. The data points are represented as a line graph, illustrating the trend of cell viability across the concentration range. Statistical significance is denoted by asterisks: *p < 0.05, **p < 0.001, and ***p < 0.0001.

The viability of IPEC-J2 cells was evaluated following co-cultivation with the *P. hominis* PHGD strain at varying concentrations and time points. The results, as depicted in Figure 4, revealed a decrease in cell viability with an increase in the concentration of *P. hominis* and extension of the co-cultivation period. Specifically, when IPEC-J2 cells were initially inoculated with 5 × 10^6^ cells/mL and 1 × 10^7^ cells/mL of *P. hominis*, a substantial reduction in the viability of IPEC-J2 cells was observed after 12, 18, and 24 h of co-incubation, compared with the control group. However, when the initial inoculation concentration was 1 × 10^6^ cells/mL of *P. hominis*, a significant decrease in the viability of IPEC-J2 cells was only observable after 18 h of co-incubation. These findings suggested that the *P. hominis* PHGD strain exerts an influence on the viability of IPEC-J2 cells in a concentration and time-dependent manner.

### Changes in mRNA expression levels of related genes in IPEC-J2 cells induced by the *P. hominis* PHGD strain

**Figure 5:**
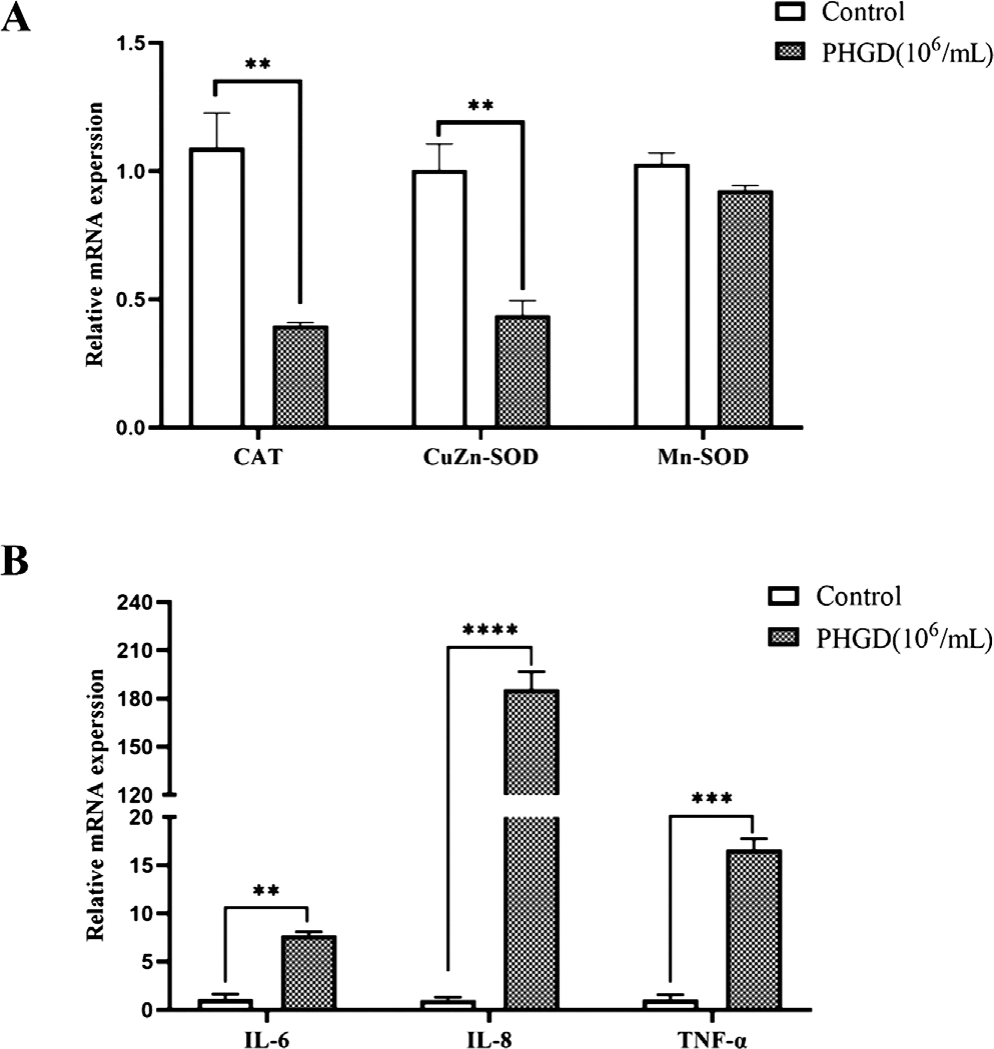
Alterations in mRNA expression in IPEC-J2 cells induced by *P. hominis*. This figure illustrates the alterations in mRNA expression of specific genes in IPEC-J2 cells following *P. hominis* induction. (A) displays changes in mRNA expression of anti-oxidative stress-related genes, while (B) presents changes in mRNA expression of inflammatory-related genes. The significance of the differences between the control group and the *P. hominis*-induced group is indicated by asterisks: *p < 0.05, **p < 0.001, and ***p < 0.0001.

The mRNA expression levels of various genes associated with cell damage and inflammation in IPEC-J2 cells were examined following co-cultivation with the *P. hominis* PHGD strain (Figure 5 and Table S1). The results revealed significant upregulation in the expression levels of IL-6, IL-8, and TNF-α within the co-cultivation group compared to the control group (*p* < 0.05). Conversely, the expression levels of CAT and CuZn-SOD experienced significant downregulation (*p* < 0.05). These genes play pivotal roles in inflammatory responses, apoptosis, and cell survival. The upregulation of inflammatory genes and the concurrent downregulation of anti-oxidative stress genes indicate that the *P. hominis* PHGD strain can trigger inflammation within IPEC-J2 cells. These findings provide valuable insights into the mechanisms through which *P. hominis* may potentially induce cellular damage and trigger inflammatory responses in the context of IPEC-J2 cell co-cultivation.

## Discussion

*Pentatrichomonas hominis* is a parasitic protozoan known to be endemic in various regions and displays a lack of host specificity. It primarily parasitizes the digestive tracts of its hosts. The pathogenicity of *P. hominis* has long been a subject of debate. In the past, it was considered a symbiotic or conditionally pathogenic entity within the intestines of both humans and animals. However, recent studies have reported the presence of *P. hominis* in the feces of affected individuals and animals [2, 3, 6, 24].

One pivotal study conducted by Chudnovskiy *et al*. [5] demonstrated that *Tritrichomonas musculis*, a newfound member of the Trichomonadaceae, contributes to the development of enteritis and colon cancer. Notably, a high infection rate of *P. hominis* (41.54%) was reported in patients with gastrointestinal cancer in China, significantly surpassing the 9.15% infection rate observed in the general population [36]. A more in-depth analysis of the gut microbiota in colon cancer patients with and without *P. hominis* infection, utilizing 16S rRNA high-throughput sequencing, revealed a higher abundance of colon cancer-related bacteria in *P. hominis*-infected individuals [35]. Despite these associations, the causal relationship between *P. hominis* infection and gastrointestinal cancer remains uncertain, necessitating further research to establish a potential causal link.

Moreover, *P. hominis* has also been identified as the causative agent of diarrhea in dogs and cats, which are often companions to humans [3, 24, 26]. Consequently, the study of *P. hominis* pathogenicity has gained increasing attention. As extracellular parasitic pathogens, trichomonads such as *P. hominis* can adhere to epithelial cells, interact with the host immune system, and influence the microbiota composition. Fang *et al*. [8] significantly contributed to our understanding of *P. hominis* in response to a zoonotic emergency through multi-omics research, characterizing hydrogenosomal proteins using RNA sequencing and proteomics techniques. Handrich *et al*. [16] employed RNA-Seq technology to confirm that the BspA and Pmp gene families are associated with enhanced adhesion of trichomonads to host cells, including *P. hominis*. Experimental evidence has shown that one *T. vaginalis* TvBspA-like and two TvPmp-like proteins promoted interactions between *T. vaginalis* and vaginal epithelial cells. Additionally, Bailey and Hirt [2] emphasized the interplay between trichomonads and the mucosal bacteria, highlighting its impact on overall health. These parasitic entities engage in competition with the intestinal microflora for adhesion with the host’s epithelial cells. Once these connections are established, the parasites can effectively evade host immune response, leading to successful colonization and subsequent infection, potentially resulting in host disease [9].

Our findings revealed that IPEC-J2 cells can facilitate the growth and proliferation of *P. hominis in vitro* following the interaction between *P. hominis* and IPEC-J2 cells. Furthermore, *P. hominis* displayed a propensity to adhere to cells and induce a degree of cellular damage. Previous studies have demonstrated that the *d* growth of *T. vaginalis* is enhanced in the presence of eukaryotic cells, with cell culture surpassing clinical broth culture [13]. Upon co-incubation, *P. hominis* exhibited adherence to IPEC-J2 cells. Intriguingly, an increase in parasite density resulted in a decrease in the adhesion rate, suggesting the presence of competitive adhesion dynamics among the parasites. With extended co-incubation time, the activity and number of IPEC-J2 cells decreased, resulting in a decrease in the adhesion rate of *P. hominis* to these cells. Notably, trichomonads can modulate the expression of its proteins after adhering to host cells, allowing it to adapt to the current living conditions. This phenomenon also impacts the virulence of trichomonads. Martínez-Herrero *et al.*’s research found that virulent strains tend to possess a greater abundance of membrane proteins and cell adhesion-related proteins, whereas the proteins of attenuated strains are predominantly associated with carbohydrate metabolism [29].

The intricate network of parasite-microbiome-host interactions involving trichomonads is characterized by their adherence to host cells, leading to severe pathological manifestations. These interactions, as observed in the case of *P. hominis*, result in mucosal ecological imbalances and, consequently, potentially benefit the trichomonads themselves [17]. Similar to previous studies on *T. vaginalis* and *T. foetus* [34], *P. hominis* has also demonstrated the ability to adhere to target cells and induce significant monolayer contraction. Trichomonad adhesion has the potential to induce alterations in mitochondrial membrane potential, resulting in substantial cytotoxicity. The cytotoxicity levels induced by different strains of trichomonads vary among clinical isolates, indicating that the ability to adhere and damage host cells is strain-dependent [21]. Our results revealed that *P. hominis* can adhere to host cells and induce a certain degree of cellular damage. However, there is no strict linear correlation between the adhesion capability and the damage potential of *P. hominis* for host cells. It should be emphasized that only when a sufficient number of *P. hominis* adhere to cells can they inflict cellular damage. This supports the notion that adhesion alone is not sufficient to cause cell damage, and it is more likely attributed to different specific effectors among the strains [28].

Our exploration of mRNA expression levels related to inflammation in IPEC-J2 cells, specifically the increased expression of pro-inflammatory cytokines (IL-6, IL-8, and TNF-α) in response to *P. hominis*, is placed within a broader context illuminated by relevant research. Hong *et al*. [18] investigated the induction of IL-6 production by *T. tenax* on oral and pulmonary epithelial cells, revealing parallels with our observations of *P. hominis*-induced inflammation. Additionally, Han *et al*. [15] delved into signaling pathways associated with IL-6 production and epithelial-mesenchymal transition in prostate epithelial cells stimulated by *T. vaginalis*, providing insights into the molecular mechanisms underlying inflammation during microbial infections. Moreover, studies have highlighted the contribution of the vaginal microbiota to trichomonad-induced inflammatory responses. Fiori *et al*. [11] demonstrated that the presence of *Mycoplasma hominis* in *T. vaginalis* isolates synergistically upregulates proinflammatory cytokines, aligning with our findings. Fichorova *et al*. [10] explored intricate interactions among vaginal microorganisms, emphasizing the collective impact of *T. vaginalis* and bacterial vaginosis on inflammation and adverse reproductive outcomes. Collectively, these studies substantiate our investigation into the interplay between microbial infections, inflammation, and immune regulation, underscoring the broader significance of comprehending host-pathogen interactions across diverse cellular contexts. Subsequent research could further delve into the regulatory roles of the gastrointestinal microbiota and *P. hominis* in modulating inflammatory responses.

The generation of reactive oxygen species (ROS) is a critical factor in inducing apoptosis in mammalian cells following infection with various pathogens [14]. Quan *et al*. [32] reported that *T. vaginalis* induces mitochondrial ROS production in SiHa cells through adhesion and infection in a time-dependent manner, thereby promoting apoptosis. In our study, the expression of anti-oxidative stress-related genes, such as CAT and CuZn-SOD, was significantly down-regulated following co-incubation of *P. hominis* with IPEC-J2 cells. This observation further confirms that *P. hominis* can induce certain damage in IPEC-J2 cells.

In conclusion, our study confirms that *P. hominis* can adhere to and rely on IPEC-J2 cells for growth and proliferation. Additionally, *P. hominis* can affect IPEC-J2 cells to varying degrees, leading to alterations in cell morphology and reductions in cell viability. The co-incubation of IPEC-J2 cells with *P. hominis* resulted in the down-regulation of anti-oxidative stress-related genes and the up-regulation of inflammatory-related genes. Therefore, we assert that *P. hominis* infection can damage intestinal epithelial cells and trigger associated inflammatory responses. These findings provide evidence for the pathogenic potential of *P. hominis* and serve as a baseline for further exploration of its pathogenesis. Further *in vitro* and *in vivo* studies are needed to validate our findings and may potentially reshape the current understanding of *P. hominis* infection.

## Supplementary material

The supplementary material for this article can be found at https://www.parasite-journal.org/10.1051/parasite/2024014/olm.

*Table S1*: Comprehensive QPCR results for alterations in mRNA expression in IPEC-J2 cells induced by *P. hominis.*
