# Supplementary material for: Unraveling the pathogenic potential of the Pentatrichomonas hominis PHGD strain: impact on IPEC-J2 cell growth, adhesion, and gene expression
Source: Parasite. 2024 Mar 26;31:18. doi: 10.1051/parasite/2024014 (PMC10964850; doi:10.1051/parasite/2024014)
Supplement: Supplementary file 1 — Table S1: Comprehensive QPCR results for alterations in mRNA expression in IPEC-J2 cells induced by P. hominis. [file parasite-31-18-s1.pdf]

Table S1. Comprehensive QPCR results for alterations in mRNA expression in IPEC-J2 cells induced by *P. hominis*

	CAT	CuZn-SOD	Mn-SOD	IL-6	IL-8	TNF-α
Control	1.09±0.14	1.01±0.10	1.03±0.04	1.13±0.52	1.04±0.28	1.10±0.47
PHGD (10^6/mL)	0.40±0.01	0.44±0.06	0.93±0.02	7.74±0.35	185.79±10.93	16.66±1.10

Note: The data includes mean ± SD values for each gene.
